# Anti-*Helicobacter pylori* activity of acomplex mixture of *Lactobacillus paracasei* HP7 including the extract of *Perilla frutescens var. acuta* and *Glycyrrhiza glabra*

**DOI:** 10.1186/s42826-020-00073-x

**Published:** 2020-10-28

**Authors:** Hyun-A Lee, Joo-Yun Kim, Jisoo Kim, Bora Nam, Okjin Kim

**Affiliations:** 1 Center for Animal Resources Development, Wonkwang University, 460 Iksandae-ro, Iksan, 54538 Republic of Korea; 2 R&BD Center, Korea Yakult Co., Ltd., 22 Giheungdanji-ro 24 beon-gil, Yongin-si, Gyeonggi-do 17086 Republic of Korea

**Keywords:** *Lactobacillus paracasei*, HP7, *Helicobacter pylori*, *Perilla frutescens var. acuta*, *Glycyrrhiza glabr*

## Abstract

The effect of standard therapeutic strategies on *Helicobacter pylori* infection is diminished over time owing to the emergence of drug resistant strains. In this study, we would like to confirm the enhanced effect of *L. paracasei* HP7, which has been reported to exert antibacterial and gastric mucosal protective effects, in combination with *Perilla frutescens* var. acuta (*P. frutescens*)and *Glycyrrhiza glabra* (*G. glabra*) extracts.

*P. frutescens* extract and *G. glabra* extract were found to inhibit the growth of *H. pylori* in a concentration-dependent manner, and the combination of *L. paracasei* HP7 and *P. frutescens* extract and *G. glabra* extract effectively inhibited *H. pylori* from attaching to AGS a gastric epithelial cells. Moreover, *L. paracasei* HP7 complex mixture containing *P. frutescens* and *G. glabra* extracts has been shown to inhibit *H. pylori* virulence genes such as AlpA, CagA, FlaA and UreA. When *H. pylori*-infected mice were administered a complex mixture of *L. paracasei* HP7 containing *P. frutescens* and *G. glabra* extract, the infection rate of *H. pylori* was significantly reduced. In addition, the *L. paracasei* HP7 complex mixture significantly reduced serum IL-8 levels and stomach inflammation in *H. pylori* infected mice.

These results suggest that a complex mixture of *L. paracasei* HP7 containing *P. frutescens* and *G. glabra* extracts may be an alternative to treating diseases caused by *H. pylori* infection.

## Introduction

*Helicobacter pylori*, a major causative pathogen of chronic gastritis [1] and gastric ulcers [2], is a spiral of gram-negative bacteria associated with an increased risk of gastric cancer [3, 4]. Vaccination with antibiotics to remove gastric *H. pylori* can reduce *H. pylori*-associated gastrointestinal diseases [5, 6] and reduce the risk of gastric cancer [7]. The standard recommended therapy for *H. pylori* uses two antibiotics, usually a triple combination therapy, including clarithromycin and a proton pump inhibitor with amoxicillin or metronidazole [8, 9]. However, the efficacy of the triple therapy has currently reduced over time. Recent cure rates of less than 80% are mainly due to the increased prevalence of resistant *H. pylori* strains in metronidazole and clarithromycin [10–12]. In addition, some patients showed allergic side effects to antibiotics and can sometimes cause side effects if *H. pylori* is not treated [13]. Long-term vaccination with antibiotics is not recommended for the prevention of *H. pylori* infection. Therefore, it is important to develop new non-antibacterial agents for the treatment of *H. pylori* [14].

*Lactobacillus spp*. is recommended as an additive to the standard recommended treatment for *H. pylori* treatment, and it is possible to improve the patient’s adaptability by reducing the side effects of antibacterial agents [15, 16]. In our previous study, we reported that the lactic acid bacterium *Lactobacillus paracasei* HP7 (*L. paracasei* HP7) isolated from kimchi, a fermented vegetable dish widely consumed in Korea, had inhibitory effects against *H. pylori* in-vitro and in-vivo [17].

Recently, there has been a clear increase in demand for natural compounds from plant extracts that are effective antibacterial agents against a wide range of bacteria to control human infection and for the preservation of food [18]. Recently, the inhibitory effect of *Glycyrrhiza glabra* (*G. glabra*) on *H. pylori* and the therapeutic effect on infected patients have been reported [19–21]. In addition, antibacterial activities [22–24] and anti-inflammatory [25–27] effects of *Perilla frutescens var. acuta* (*P. frutescens*) have been reported.

In this study, we aimed to determine whether the combination of *L. paracasei* HP7 and *P. frutescens* and *G. glabra* extracts had a synergistic effect on the inhibition of *H. pylori* infection.

## Methods/experimental

### Bacterial strains

*L. paracasei* HP7 was incubated at Man-Rogosa-Sharpe broth (Difco Laboratories, Detroit, Mich.) at 35 °C for 24 h. *H. pylori* strain SS1 (B0890; Korean Jeongeup Korean Collection) was cultured overnight at 37 °C. under microaerobic conditions in brain-heart infusion medium containing 10% fetal bovine serum (FBS) and grown to density ~ 2.0 × 10^9^ CFU/mL. The cultured bacteria were then transferred to phosphate buffered saline (PBS) before the test.

### Herbal extract

Each of the herbal extracts of *G. glabra* and *P. frutescens* were obtained from Korea Yakult Co., Ltd.

### Cell culture

Human gastric cell line AGS cells (human gastric adenocarcinoma) were obtained from the Korean Cell Line Bank (cellbank.snu.ac.kr) and used. For maintenance and proliferation of cells, passage was performed every 2 days at 37 °C and 5% CO_2_ using Ham’s F-12 medium containing 10% FBS and 1% antibiotic. For analysis of *H. pylori* infection to gastric cells, antibiotics were not added to the culture medium.

### *H. pylori* growth inhibition

To confirm the anti-*H.pylori* activity of *P. frutescens* and *G. glabra* extracts, Alamar blue assay was performed by referring to the study of Tsukasa M et al. [28]. *H. pylori* was suspended in DMEM / F-12 containing 5 mM L-lactic acid with a turbidity of 0.005 (1 × 10^5^ CFU/mL). One hundred microliter suspension was added to 96 well culture plate and then incubated for 4 h at 37 °C with the test material (*P. frutescens* and *G. glabra* extracts) under micro-aerophilic conditions. After incubation, inhibition of *H. pylori* growth was measured by Alamar blue according to manufacturer’s criteria (Alamar Bio-Sciences, Sacramento, CA, U.S.A.). *H. pylori* inhibitory activity of the tested material was calculated by the following formula:

$$ \mathrm{inhibition}\ \left(\%\right)=\left[\left(\mathrm{A}-\mathrm{B}\right)/\left(\mathrm{A}-\mathrm{C}\right)\right]\times 100 $$

A: cultured without test sample. B: cultured with test sample. C: medium alone.

### Inhibition of *H. pylori* adhesion to AGS cells

AGS cells were cultured in 6-well plates for 16 h. When the cells reached 90% confluence, the medium was replaced with serum and antibiotics-free F-12 medium. An overnight cultured *H. pylori* SS1 was suspended in Ham’s F-12 medium. For co-culture of bacteria and gastric epithelial cells, *H. pylori* SS1 (10^7^ CFU) were added to wells containing 10^6^ AGS cells and incubated for 4 h in the absence or presence of herbal extracts and *L. paracasei* HP7. The adhesion of *H. pylori* was measured using a Real-Time PCR system (Applied Biosystems, Foster City, CA, USA) as in our previous paper [17]. Forward and reverse sequences of primers for amplifying the *H. pylori* 16S RNA gene were as follows: 5′-TCG GAA TCA CTG GGC GTA A-3′ and 5′-TTC TAT GGT TAA GCC ATA GGA TTT CAC-3′.

### Detect of *H. pylori* virulence gene expression

*H. pylori* SS1 cells were cultured in brain-heart infusion broth at ~ 1.0 × 10^7^ CFU/mL. Cultured *H. pylori* were treated with *G. glabra* extract (3μg / mL), *P. frutescens* extract (25μg/mL), and *L paracasei* HP7 (1.0 × 10^7^ CFU/mL) and incubate at 37 °C for 2 h. cDNA was synthesized using murine leukemia virus reverse transcriptasptase with random hexamer. Primer sequence for *H. pylori* virulence genes are listed in Table 1. AlpA is genes that *H. pylori* attaches to the gastric mucosa, and CagA plays the role of *H. pylori* invading gastric cells. FlaA is related to the mobility of *H. pylori*, and UreA is genes that *H. pylori* uses to neutralize gastric acid [29].

**Table 1:** PCR primer sequence for *H. pylori* virulence genes

Gene name	Sequence	Tm (° C)	Reference
alpA	F: AAACCGCTCTGTGGATATGG	55.0	NZ_CP009259.1
	R: GAACTGGAAGTGCGTGTTATTG	45.6	
cagA	F: TCACTCTTGGCGATATGGAAAT	57.5	
	R: ACACAGAAGACAGAGCGTTATT	57.7	
flaA	F: GCTAAGAGCATCAATGTGGTTTC	58.3	
	R: CGGTAACATCGCGCAAATTC	58.5	
ureA	F: AGTGGGTATTGAAGCGATGTT	57.6	
	R: AAGAACAACTCACCAGGAACTAA	57.6	

### Animals

Specific pathogen free (SPF) male C57BL/6 mice weighing 20–24 g were purchased from Samtako Co. (Osan, Korea) and were maintained at the inspection facility of Wonkwang University (Iksan, Korea) for 1 week before experiments. Thereafter, the mice were maintained in an SPF barrier room with regulated temperature (23 °C ± 1 °C) and humidity (50% ± 5%) and a 12:12-h light/dark cycle. The animals were fed a sterilized pellet diet (Purina, Seoul, Korea) and sterilized water ad libitum. All studies were performed in accordance with the Guide for Animal Experimentation of Wonkwang University and were approved by the Institutional Animal Care and Use Committee of Wonkwang University (approval no. WKU 2019-08-22).

### *H. pylori* inoculation

Animals were intragastrically inoculated three times, with a 3-day interval between inoculations, with *H. pylori* at ~ 1.0 × 10^9^ CFU in 0.5 mL broth. The challenged animals were confirmed as *H. pylori*-positive by stool antigen analysis using the Bioline *H. pylori* Ag kit (Standard Diagnostics, Suwon City, Korea) as previously described [30].

### In vivo study protocol

The inhibition of *H. pylori* growth by *L. paracasei* HP7 was investi in a mouse model. The mice were divided into six groups: negative control (NC, *n* = 10); *H. pylori*-infected without treatment (C, *n* = 10); *H. pylori*-infected with positive control Deglycyrrhizinated Licorice (DGL) [20] treatment (D, *n* = 10); *H. pylori*-infected with *P. frutescens* extract (PFE) 5 mg/kg + *G. glabra* extract (GGE) 1.2 mg/kg (COM 1, n = 10); *H. pylori*-infected with PFE 10 mg/kg + GGE 1.2 mg/kg (COM 2, n = 10); and *H. pylori*-infected with *L. paracasei* HP7 2.0 × 10^7^ CFU + PFE 10 mg/kg + GGE 1.2 mg/kg (COM 3, n = 10). All substances were administered orally once daily for 4 weeks. At the end of the experiment, the animals were euthanized with ether, and then dissected. The stomach was further incised along the taiwanese valley, and washed with saline. The remaining portion was formalin fixed and inserted into paraffin for histological analysis. *H. pylori* colonies were confirmed by the aforementioned quick urease test (CLO-test) [30].

### Blood analysis

Blood samples were collected from the hearts of sacrificed animals,centrifuged at 1000×*g* for 15 min at 4 °C, and the isolated plasma was stored at − 80 °C. Serum titers of anti-*H. pylori* antibodies were measured using a mouse anti-*H. pylori* antibody (IgG-1) ELISA kit (Cusabio Biotech, Wuhan, China) in accordance with the manufacturer’s instructions. IL-8 levels in mice were measured using the Mouse Interleukin 8 ELISA Kit (R&D System, Minneapolis, USA) in accordance with the manufacturer’s instructions.

### Statistical analysis

Experimental results were compared between groups using Minitab (State College, PA, USA) and one-way ANOVA, a parametric multiple comparison procedure. The results were expressed as mean ± standard error and statistically significant when *P* < 0.05.

## Results

### *H. pylori* growth inhibition

We measured the *H. pylori* growth inhibitory activity of 140 plant extracts including *G. glabra* and *P. frutescens*. Excluding non-edible plants, *G. glabra* and *P. frutescens* extracts showed the best inhibitory effect on the growth of *H. pylori*. In particular, *G. glabra* 90% ethanol extract and *P. frutescens* 50% ethanol extract showed high activity (data not shown).

**Fig. 1:**
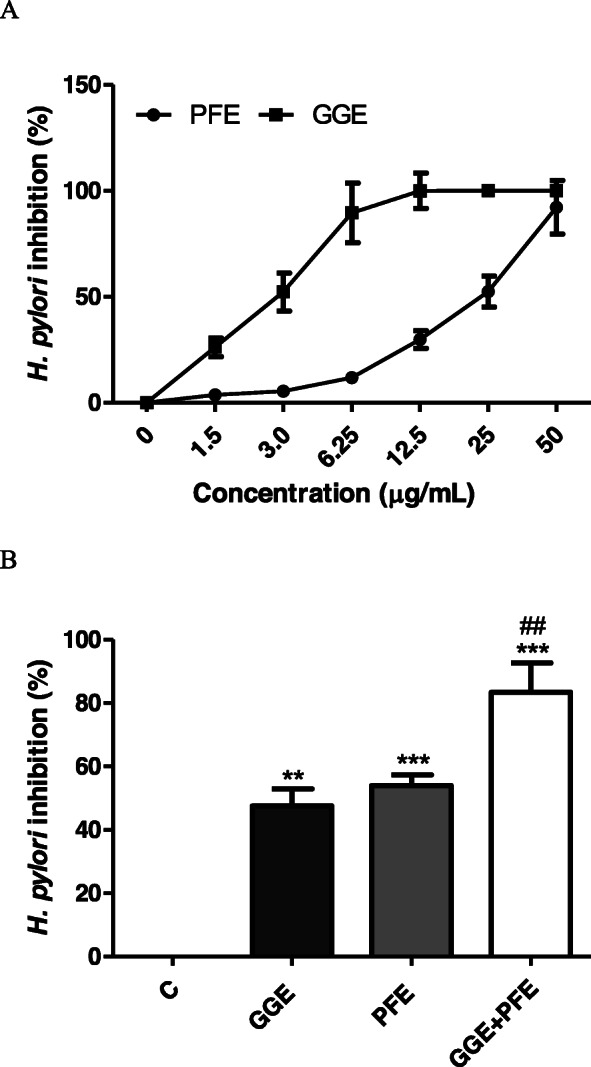
Inhibitory effects of *P. frutescens* and *G. glabra* extract against growth of *H. pylori.* **a** *H. pylori* growth inhibitory activity of *P. frutescens* and *G. glabra* extracts at various concentrations. **b** *H. pylori* inhibitory activity following co-treatment of *P. frutescens* and *G. glabra* extract. PFE, *P. frutescens* extract; GGE, *G. glabra* extract. **Significantly different from the non treated control C (*P* < 0.01). ***Significantly different from the non treated control C (*P* < 0.001). ##Significantly different from the PFE and GGE treated group (*P* < 0.01)

There have been several reports of antibacterial and Helicobacter pylori inhibitory activity of *P. frutescens* and *G. glabra* [19–24]. However, there have been few reports of synergistic effects of *H. pylori* inhibitory activity of *P. frutescens* and *G. glabra*. Therefore, the *H. pylori* growth inhibitory activity of each of the *P. frutescens* extract (PFE) and *G. glabra* extracts (GGE) was investigated, and whether the two extracts had a synergistic effect on *H. pylori* inhibition was examined. PFE and GGE inhibited the growth of *H. pylori* in a concentration-dependent manner. PFE and GGE almost completely inhibited the growth of *H. pylori* at concentrations of 12.5 μg/mL and 50 μg/mL, respectively, and the IC_50_ of each extract was 23.84 μg/mL and 2.88 μg/mL. (Fig. 1a). When the extract corresponding to IC_50_ was co-treated, the growth of *H. pylori* was inhibited by about 90% (Fig. 1b). This suggests that *P. frutescens* and *G. glabra* are synergistic in inhibiting the growth of *H. pylori*.

### Suppression of *H. pylori* adhesion to gastric epithelial cells

**Fig. 2:**
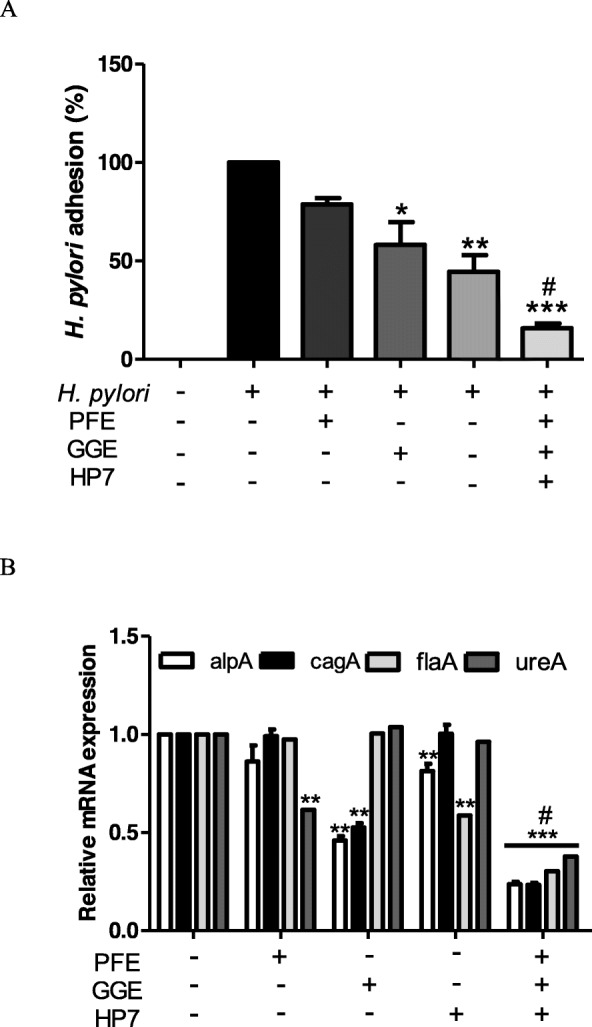
Effect of complex mixture of *L. paracasei* HP7 containing *P. frutescens* and *G. glabra* extract on *H. pylori* adhesion and *H. pylori* virulence genes expression (**a**) Degree of *H. pylori* attached to AGS cells (**b**) alpA, cagA, flaA and ureA mRNA expression in *L. paracasei* HP7, *P. frutescens* extract, *G. glabra* extract and complex mixture treated *H. pylori* SS1. PFE, *P. frutescens* extract 25 μg/mL; GGE, *G. glabra* extract 3 μg/mL; HP7, *L. paracasei* HP7 1.0 × 107 CFU/Ml. *Significantly different from non-treated control C (*P* < 0.05). **Significantly different from the non-treated control C (*P* < 0.01). ***Significantly different from the non-treated control C (*P* < 0.001). #Significantly different from the PFE, GGE and HP7 treated group (*P* < 0.05)

In a previous study, we confirmed that hp7 inhibits Helicobacter pylori adhesion to gastric epithelial cells [17]. The complex mixture of *L. paracasei* HP7 containing PFE and GGE significantly inhibited *H. pylori* adhesion to gastric cells than *L. paracasei* HP7 or PFE or GGE alone (Fig. 2a). These results demonstrate that *L. paracasei* HP7 and *P. frutescens* and *G. glabra* extracts are synergistic in inhibiting bacterial adhesion to gastric epithelial cells.

### Inhibition of *H. pylori* virulence factor

*H. pylori* produces urease to decompose the urea in the stomach, reduce the acidity around it, move using flagella, and attach to epithelial cells through adhesion factors such as AlpA. In addition, the CagA protein secreted by *H. pylori* inflames gastric epithelial cells and causes gastric cell changes known as the “hummingbird phenomenon” [1, 2, 29]. Therefore, we investigated the effect of a complex mixture of *L. paracasei* HP7 containing PFE and GGE on the mRNA expression of genes encoding AlpA, Cag, FlaA, and UreA of *H. pylori.*

PFE significantly reduced ureA and GGE decreased alpA and cagA. HP7 significantly reduced flaA associated with *H. pylori* motility. Meanwhile, the HP7 complex mixture significantly reduced *H. pylori* virulence genes compared to PPE or GGE or HP7 alone (Fig. 2b).

### *A*nti-*H. pylori* antibody titer in serum

To confirm the colonization of *H. pylori* in mice, the absorbance of IgG serum against *H. pylori* was also related to *H. pylori* colonization, so anti-Helicobacter IgG-1 serum levels were measured [31]. The serum antibody titers were elevated 4 weeks after *H. pylori* inoculation, to values of 1.48 ± 0.06, 0.94 ± 0.07, and 0.95 ± 0.04 in the *H. pylori* infection (Group C), positive control DGL (Group D), and *H. pylori* infection/*L. paracasei.*

**Fig. 3:**
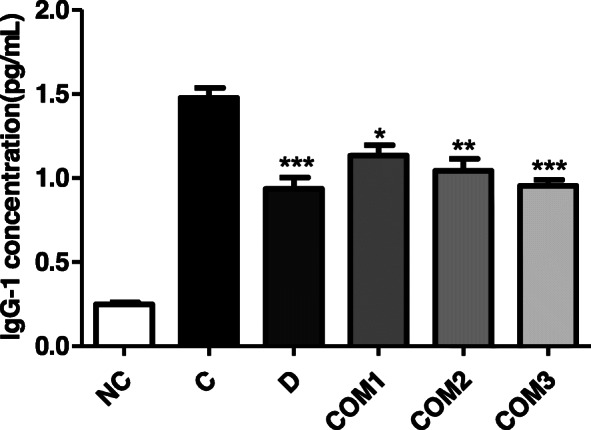
Results of *Helicobacter pylori* antibody (IgG) test with mice serum. *Significantly different from the infection control Group C (*P* < 0.05). **Significantly different from the infection control Group C (*P* < 0.01). ***Significantly different from the infection control Group C (*P* < 0.001)

HP7 + PPE + GGE (Group COM3) treatment groups, respectively, as compared with 0.25 ± 0.01 in control animals (Group NC) (Fig. 3).

These results indicated that *H. pylori* infection was significantly reduced by treatment with a complex mixture of *L. paracasei* HP7 containing extracts of PPE and GGE.

### Decrease of *H. pylori* colonization

Repeated intragastric inoculation of C57BL/6 mice treated with *H. pylori* (1.0 × 10^9^ CFU/mouse, three times) led to a positive reaction in the gastric mucosal campylobacter-like organism (CLO) test (Table 2). Positive percentages were increased 4 weeks after *H. pylori* inoculation, with values of 100% (CI 72.2–100), 30% (CI 10.8–60.3), 10% (CI 1.8–40.4) in the *H. pylori* infection (Group C), positive control DGL (Group D) and *H. pylori* infection/*L. paracasei* HP7 + PPE + GGE (Group COM3) treatment groups, respectively, compared with 0% (CI 0–27.6) in control animals (Group NC) (Table 2).

**Table 2:** Reactivity in the CLO test of gastric mucosa from mice infected with *H. pylori* followed by treatment with *L.* HP7 and herbal extracts

Group	Treatment	n	Positive %^a^	Therapeutic %
NC	Normal control	10	0%, CI^b^0–27.6	100%,CI72.2–100
C	*H. pylori*	10	100%, CI 72.2–100	0%, CI 0–27.6
D	*H. pylori* + DGL	10	30%, CI 10.8–60.3	70%, CI 39.7–89.2
COM1	*H. pylori* + PPE5 + GGE1.2	10	50%, CI 23.7–76.3	50%, CI 23.7–76.3
COM2	*H. pylori* + PPE10 + GGE1.2	10	30%, CI 10.8–60.3	70%, CI 39.7–89.2
COM3	*H. pylori* + HP7+ PPE10+ GGE1.2	10	10%, CI 1.8–40.4	90%, CI 60.0–98.2

*DGL* Deglycyrrhizinated Licorice, *HP7 L. paracasei* HP7, *PPE P. frutescens var. acuta* extract, *GGE Glycyrrhiza glabra* extract

^a^A positive percentage reflects *H. pylori* colonization, which was observed as medium color change from yellow to red

^b^Incidence (95% confidential interval [CI]) was calculated using MiniTab statistical software

**Fig. 4:**
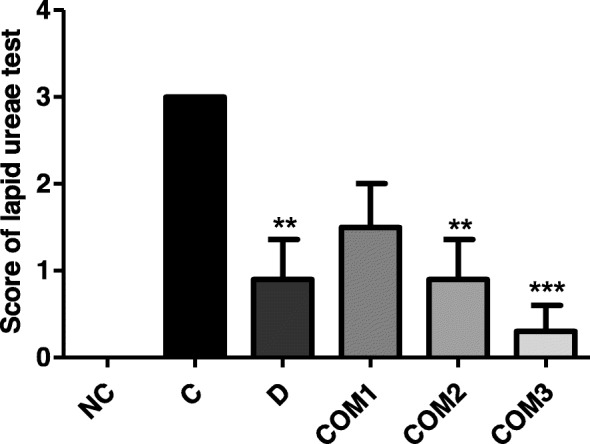
Reactivity in the CLO test of gastric mucosa from mice infected with *H. pylori* followed by treatment with complex mixture of *L. paracasei* HP7 and herbal extracts. **Significantly different from the infection control Group C (*P* < 0.01). ***Significantly different from the infection control Group C (*P* < 0.001)

CLO scores were decreased by *H. pylori infection*/*L. paracasei* HP7 + PPE + GGE (Group COM3) relative to *H. pylori*-infected animals without treatment (Group C) (*P* < 0.01; Fig. 4). Therefore, *L. paracasei* HP7 + PPE + GGE may reduce the colonization rate of *H. pylori*.

### Alleviation of gastric mucosal lesions caused by *H. pylori*

**Fig. 5:**
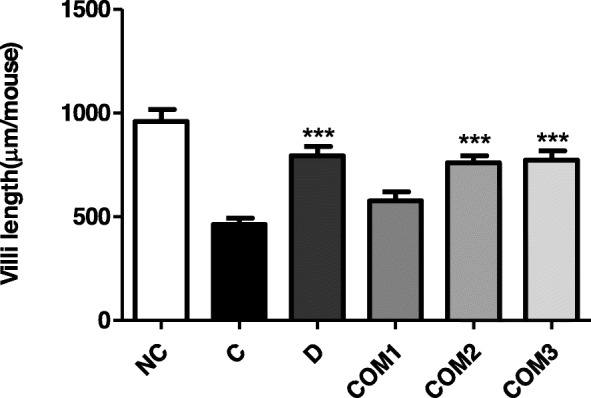
Gastric mucosal viili lengths of mice infected with *H. pylori* followed by treatment with complex mixture of *L. paracasei* HP7 and herbal extracts. ***Significantly different from the infection control Group C (*P* < 0.001)

Pathological changes in the gastric mucosa were minimal in animals not infected with *H. pylori* (Group NC). In contrast, Group C (*H. pylori* inoculated) mice exhibited gastric atrophy and severely shortened villi. However, mice in Group COM3 (*H. pylori* infected/*L. paracasei* HP7 + PPE + GGE) showed a significant improvement in gastric mucosa. These results were confirmed by an increase in villus length in Group COM3 compared with Group C (Fig. 5).

### Suppression of *H. pylori*-induced IL-8 production

**Fig. 6:**
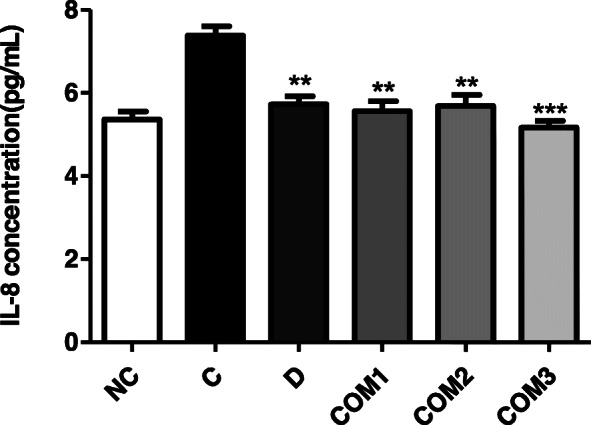
Serum IL-8 levels of mice infected with *H. pylori* followed by treatment with complex mixture of *L. paracasei* HP7 and herbal extracts. **Significantly different from the infection control Group C (*P* < 0.01). ***Significantly different from the infection control Group C (*P* < 0.001)

Blood IL-8 levels were elevated 4 weeks after *H. pylori* inoculation, with values of 7.39 ± 0.70, 5.73 ± 0.63, 5.16 ± 0.49 in the *H. pylori* infection (Group C), positive control DGL (Group D) and *H. pylori* infection/*L. paracasei* HP7 + PPE + GGE (Group COM3) treatment groups, respectively, as compared to 5.36 ± 0.59 in control animals (Group NC) (Fig. 6).

## Discussion

Lactic acid bacteria suppress the growth of human bacterial pathogens by secreting compounds, such as antibiotics, organic acids, and bacteriocins, to lower the pH of the environment and control gastrointestinal infections [31, 32]. The inhibitory activity of *H. pylori* has been reported in several *Lactobacillus spp*., including *L. acidophilus* [32], *L. casei* [33], *L. johnsonii* [34], *L. reuteri* [35], and *L. salivarius* [36].

A new *Lactobacillus spp*. isolated from kimchi by Korea Yakult Co. Ltd. was identified as *L. paracasei* and was named strain HP7. Kimchi is considered a healthy food as it is enriched in vitamins A, B, and C, and is high in fiber, but also contains a number of lactic acid bacteria [37]. The two herbs selected in this study were *P. frutescens var. acuta* and *G. glabra*, which showed the strong antibacterial activity of *H. pylori* by measuring the Helicobacter antibacterial activity (growth suppression) in the extraction of natural product candidates through the inhibitory clear zone test of *H. pylori* (data not shown).

*G. glabra* (licorice) was reported to exhibit antimicrobial activity against several gram-negative and gram-positive bacterial strains including *H. pylori* [38]. In addition, licorice also exerted beneficial effects against *H. pylori* through its antiadhesive properties [39]. Activity against ulcer and cancer, and clinical outcomes of *H. pylori* infection were also exhibited by licorice. The curative effect of deglycyrrhizinated licorice (DGL) on ulcers has been reported in vivo and in clinical studies [40–42], and the anticancer effect of licorice extract was shown in an in vitro study [43]. *G. glabra* was shown to possess anti-ulcerogenic properties that may be conferred by the cytoprotective mechanism of its antioxidant properties. These results supported the ethnomedical uses of licorice in the treatment of gastric ulcer [44].

Traditionally, *P. frutescens var. acuta* has been prescribed to treat depression- related disease, anxiety, asthma, chest stuffiness, vomiting, cough, cold, flu, phlegm, tumors, allergies, intoxication, fever, headache, stuffy nose, constipation, abdominal pain, and indigestion, and acts as an analgesic, anti-abortive agent, and a sedative [23]. The antibacterial activity of *P. frutescens var. acuta* has also been reported [24].

In this study, we confirmed in vitro and in vivo experiments of *H. pylori* inhibitory activity of a *L. paracasei* HP7 complex mixture containing *P. frutescens var. acuta* and *G. glabra* extracts. *P. frutescens* extract and *G. glabra* extract inhibited the growth of *H. pylori* in a dose-dependent manner, and the *H. pylori* growth inhibitory effect was increased when the two extracts were mixed at IC_50_ concentration. In addition, the inhibitory effect of adhesion of gastric epithelial AGS cells of *H. pylori* by the *L. paracasei* HP7 or *P. frutescens* extract and *G. glabra* extract, when applied in a complex mixture, rather than each individually, was confirmed to be larger. Also, we confirmed the inhibitory activity of a complex mixture of *L. paracasei* HP7 including the extracts of *P. frutescens* and *G. glabra* against *H. pylori* in a mouse model; a rapid urease test of mouse stomachs showed decreased *H. pylori* colonization. Thus, the eradication of *H. pylori* reduced inflammation and epithelial damage in the stomach, although it is also possible that a complex mixture of *L. paracasei* HP7 including the extract of *P. frutescens* and *G. glabra* had direct anti-inflammatory effects on the gastric mucosa.

Although triple therapy consisting of two antibiotics and a proton pump inhibitor is effective over a short term and helps to maintain patient compliance, many patients experience undesirable side effects such as diarrhea, epigastric pain, nausea, and bloating [45].

In comparison, a complex mixture of *L. paracasei* HP7, including the extracts of *P. frutescens* and *G. glabra*, is safe and therefore appropriate for the prevention and treatment of *H. pylori* infection. In this study, the therapeutic effect of a complex mixture of *L. paracasei* HP7 including the extract of *P. frutescens* and *G. glabra*, was partial, at 90%. However, *H. pylori* adhesion and a reduced inflammatory response was shown. Other researchers reported also that probiotics alone could not completely eliminate *H. pylori*, but could reduce the load of *H. pylori* in the stomach, and alleviate gastric mucosal inflammation [46, 47]. Accumulating evidence suggests an important role of IL-8 in *H. pylori* infection-associated chronic atrophic gastritis, peptic ulcer and gastric cancer [48]. The suppression of IL-8 by a complex mixture of *L. paracasei* HP7, including the extract of *P. frutescens* and *G. glabra*, can potentially prevent *H. pylori*-induced gastritis and carcinogenesis in the stomach.

Previously, the results of our study reported that *L. paracasei* HP7 alone was able, to some extent, suppress *H. pylori* infection [17]. This study was performed to confirm the elevation effect of compounds mixed with *P. frutescens* and *G. glabra* extract, which are known to have antibacterial and gastric mucosal protective effects other than *L. paracasei* HP7.

## Conclusions

The administration of a complex mixture of *L. paracasei* HP7 containing an extract of *P. frutescens* and *G. glabra* was more effective than that of *L. paracasei* HP7 alone or *P. frutescens* extract or *G. glabra* extract, and the administration of a higher antibacterial effect of *H. pylori* and inflammation induced by *H. pylori* or it was confirmed to reduce the damage to the mucous membrane. The mechanism of this action resulted from the inhibitory effect of *L. paracasei* HP7 on the adhesion of *H. pylori* to the gastric mucosa, the antibacterial effect and antioxidative effect of *G. glabra* and *P. frutescens* extract, and the increased secretion of gastric mucosal mucin. It can be assumed that the anti-*H.pylori* effect and the protective effect on the gastric mucosa were induced. Thus, a complex mixture of *L. paracasei* HP7, including the extract of *Perilla frutescens* and *Glycyrrhiza glabra* can be used to treat patients with gastric symptoms, including ulcers caused by *H. pylori.*

These results demonstrated that treatment with a complex mixture of *L. paracasei* HP7, including the extract of *P. frutescens* and *G. glabra* could inhibit the growth of *H. pylori* and is thus a promising treatment for patients with gastric symptoms, such as gastritis, that are caused by *H. pylori* infection.

## Data Availability

I declare that this manuscript has the availability of data and material.
